# Factors Associated With Unhealthy Snacks Consumption Among Adolescents in Iran’s Schools

**DOI:** 10.15171/ijhpm.2017.09

**Published:** 2017-01-29

**Authors:** Vahid Yazdi Feyzabadi, Nastaran Keshavarz Mohammadi, Nasrin Omidvar, Akram Karimi-Shahanjarini, Saharnaz Nedjat, Arash Rashidian

**Affiliations:** ^1^ Department of Health Management and Economics, School of Public Health, Tehran University of Medical Sciences, Tehran, Iran.; ^2^ School of Public Health, Shahid Beheshti University of Medical Sciences, Tehran, Iran.; ^3^ Department of Community Nutrition, National Nutrition and Food Technology Research Institute, Faculty of Nutrition Sciences and Food Technology, Shahid Beheshti University of Medical Sciences, Tehran, Iran.; ^4^ Social Determinants of Health Research Center and Department of Public Health, School of Public Health, Hamadan University of Medical Sciences, Hamadan, Iran.; ^5^ Epidemiology and Biostatistics Department, School of Public Health, Knowledge Utilization Research Center, Tehran University of Medical Sciences, Tehran, Iran.

**Keywords:** School Health Promotion, Health Behavior, Snack, Nutrition Policy, Adolescent

## Abstract

**Background:** Well-informed interventions are needed if school-based health promotion is to be effective. Among other aims, the Iranian Health Promoting School (IHPS) program that was launched in 2011, has an important aim of promoting dietary behaviors of adolescents. The present study, therefore, aimed to investigate the factors affecting unhealthy snacking of adolescents and provide evidence for a more effective IHPS program.

**Methods:** In a cross-sectional study design, 1320 students from 40 schools in Kerman city were selected using a proportional stratified random sampling method. A modified qualitative Food Frequency Questionnaire (FFQ) was used to gather data about unhealthy snacking behavior. Data about intrapersonal and environmental factors were obtained using a validated and reliable questionnaire. A mixed-effects negative-binomial regression model was used to analyze the data.

**Results:** Taste and sensory perception (prevalence rate ratio [PRR]=1.18; 95% CI: 1.09-1.27), being a male (PRR=1.20; 95% CI: 1.05-1.38) and lower nutritional knowledge (PRR=0.96; 95% CI: 0.91-0.99) were associated with higher weekly unhealthy snaking. Perceived self-efficacy (PRR=0.95; 95% CI: 0.91-1.00) negatively influenced the frequency of unhealthy snaking, with this approaching significance (*P*<.06). In case of environmental factors, high socio-economic status (SES) level (PRR=1.45; 95% CI: 1.26-1.67), single-parent family (PRR=1.14; 95% CI: 1.01-1.30), more social norms pressure (PRR=1.08; 95% CI: 1.01-1.17), pocket money allowance (PRR=1.21; 95% CI: 1.09-1.34), easy accessibility (PRR=1.06; 95% CI:1.01-1.11), and less perceived parental control (PRR=0.96; 95% CI: 0.92-0.99) all had a role in higher consumption of unhealthy snacks. Interestingly, larger school size was associated with less unhealthy snacking (PRR=0.79; 95% CI: 0.68-0.92).

**Conclusion:** Unhealthy snacking behavior is influenced by individual, socio-cultural and physical-environmental influences, namely by factors relating to poor parenting practices, high SES level, family characteristics, improper social norms pressure, and less knowledge and self-efficacy of students. This evidence can be used to inform a more evidencebased IHPS program through focusing on supportive strategies at the home, school, and local community levels.

## Background

Unhealthy eating behaviors and habits act as risk factors for many non-communicable diseases (NCDs), including cancer, obesity, high blood pressure, cardiovascular diseases, and diabetes.^1,2^ This matter has prompted some global health alliances to plan for measures that target consumption of foods high in fat, salt, and sugar.^3^ It is believed that these behaviors and habits are mostly shaped, consolidated, or intensified during childhood and adolescence.^4^ This highlights the importance of childhood and adolescence as sensitive phases that provide great opportunities for different preventive interventions to ensure future health.^5^

Schools, as a key setting for health promotion practice, significantly contribute to development and promotion of health-related behaviors among adolescents.^6^ Health Promoting School (HPS) initiative is one of the most prominent school-based interventions proposed globally. This initiative, known as a whole-school approach, allows health to be placed on school agenda by using a collaborative approach in a school setting. It combines educational, physical, and social-driven approaches to affect determinants of health lifestyle, such as eating behaviors.^7^ Over recent years and in line with a global movement, Iran has adopted an eight-component program named as Iranian Health Promoting School (IHPS) in its schools, mainly the public ones, throughout the country. The public schools provide the minimum required infrastructure, in terms of human resources and physical environment, required for such a program.^8^ One of the components in IHPS is devised to promote dietary behaviors of students. This component focuses on limitation of unhealthy snacks consumption and promotion of healthy snacks consumption through proper educational and environmental interventions.^8^

Some studies have shown that adoption of HPS framework results in improvement of dietary outcomes such as higher intake of healthy foods, limited consumption of unhealthy foods, improvement in body mass index (BMI), and obesity reduction.^9-11^ However, systematic reviews have also reported of varying effects.^9,12^ In this regard, one of the most important issues that has received high attention is the extent to which HPS and similar interventions rely on well-informed interventions and contextual factors.^12-15^

It is evident that there have been changes in eating behaviors due to a nutrition transition over the recent decades worldwide; mainly a shift from diets high in vegetables, fruits, cereal, and fiber to diets high in sugar, salt, and fat).^16^ The transition determinants highly depended on contextual features, mainly on economic and socio-cultural factors.^17^ However, despite a plethora of studies on determinants of unhealthy diet from across the world,^18,19^ most of the relevant evidence comes from developed countries. Nevertheless, better understanding of influential factors on unhealthy snacks consumption may provide a basis for policy-makers to develop well-informed school-based health promotion interventions, such as IHPS, to target those factors. This study, therefore, aimed to identify unhealthy snacks consumption determinants among adolescents in Kerman, Iran and, consequently, provide the ground for a more comprehensive approach to plan, develop, and implement suitable and context-sensitive future interventions.

## Methods

### Participants and Sampling

In a cross-sectional study, 1320 eighth grade students from 40 secondary schools in Kerman city, located in southeast of Iran, were selected using a proportional stratified random sampling method. At first, a list of schools was collected from the respective education sector. Then, the intended schools were randomly selected from the strata defined by gender, type of school (public/private), and involvement in IHPS (IHPS/non-IHPS). In order to control for selection bias, random selection of schools was based on districts of the city that differed in terms of SES. To select participants, a list of eighth-grade students was obtained from the principals of selected schools and the students were randomly selected using random number tables. As some potential confounding factors might be present in different grades, the eighth grade students (14 years old) from middle schools were selected as the sampling frame. This study was part of a larger study to analyze IHPS program and one of its parts related to snacking behaviors differences between IHPS and non-IHPS schools is going to be reported elsewhere.

### Independent Measures

In order to determine the factors that affect unhealthy snacking behavior, potential individual and environmental factors influencing adolescents’ eating behaviors were extracted from a study conducted by Story et al.^20^ In their study, using ecological model and social cognitive theory, they conceptualize that eating behavior of adolescents is a function of individual and environmental factors. In the present study, included individual (intrapersonal) factors were as follows: gender, taste and sensory perception, perceived self-efficacy, nutritional knowledge, cost/price sensitivity, and self-body image. Environmental factors were defined as social-environmental (family and parenting characteristics), physical-environmental (eg, schools and community characteristics), and macro-system (eg, media) factors. These factors, in fact, consisted of family structure (single-parent vs. dual parent), pocket money allowance (yes/no), socio-economic status (SES), social norms pressure, perceived parental control, accessibility of unhealthy snacks at home and shops, media advertisements, and school characteristics. The main measures are separately described in the following section.

*Cost/price sensitivity.* This measure was a single-item measure in which students were asked to indicate the extent of their agreement with the item (“Because unhealthy snacks are less expensive I eat them”). This measure indicates whether students are cost-sensitive to unhealthy snacking or not. Response options ranged from strongly disagree = 1 to strongly agree = 5; the higher the score, the higher the role of cost in unhealthy snacks consumption.

*Taste and sensory perception.* This measure was assessed using 3 items (eg, “Because unhealthy snacks are tasty and delicious, I eat them”). Students were asked to indicate to what extent they agree that better taste and appeal of unhealthy snacks stimulate them to consume those foods. Response options ranged from strongly disagree = 1 to strongly agree = 5; the higher the score, the higher the role of taste and sensory perception in unhealthy snacks consumption.

*Nutritional knowledge.* This measure refers to the ability to distinguish between the most common healthy and unhealthy snacks. It was assessed using four statements (eg, “Which one of the following items is a healthy drink?”) that measured students’ knowledge about common healthy and unhealthy snacks. Response options of the statements were multiple-choice, the choice scored 1 if it was correct and 0 otherwise. Thus, the score ranged from 0 (none of the statements was correctly answered) to 4 (all four statements were correctly answered); the higher the score, the higher the nutritional knowledge level.

*Self-body image.* This measure was a single-item measure which represented the body fitness perceived by students (ranging from very thin = 1 to very obese = 5).

*Perceived self-efficacy.* This variable represented a student’s belief in her/his ability to succeed in controlling of unhealthy snacks consumption and weight gain. This was assessed using three items (eg, “How confident are you that if you wanted to, you could reduce consumption of the certain unhealthy snacks?). Response options ranged from “1” (not at all confident) to “5” (extremely confident); the higher the score, the higher the perceived self-efficacy to reduce consumption of unhealthy snacks.

*Social norms pressure*. This measure represents the pressure induced by peers, friends, family and generally society to consume unhealthy snack. This measure was assessed using seven items (eg, “Society expects me as an adolescent to eat unhealthy snacks”). Response options ranged from strongly disagree = 1 to strongly agree = 5; the higher the score, the higher the social norms pressure for more consumption of unhealthy snacks.

*Perceived parental control.* This variable was measured using three items (eg, “My parents tell me how much junk food I may consume”) that were derived from de Bruijn et al study.^21^ Response options ranged from strongly disagree = 1 to strongly agree = 5; the higher the score, the higher the perceived parental control on unhealthy snacks consumption.

*Media advertisements.* This measure was assessed using two items (eg, “Advertisement on TV or radio stimulates me to eat unhealthy snacks”). This measure indicates the extent to which students agree that advertisement on TV, radio, or local outdoor advertisement stimulate unhealthy snacks consumption. Response options ranged from strongly disagree = 1 to strongly agree = 5; the higher the score, the higher the role of media advertisements on unhealthy snacks consumption.

*Easy accessibility.* This measure represents the easiness of access to unhealthy snacks at home or in shops. The measure was measured using two items (eg, “If I had access to unhealthy snacks at home, it would make it more difficult for me to avoid these food items”). Response options ranged from strongly disagree = 1 to strongly agree = 5; the higher the score, the higher the accessibility to unhealthy snacks.

*SES level.* SES level of students was constructed using principal component analysis (PCA) method^22,23^ which has also been used in other studies about dietary behaviors.^24,25^ In our study, a composite measure was constructed from a combination of available data about family economic status (ie, housing characteristics) and parents’ education and occupation status. This combination was expected to better explain the SES level of students and minimize the risk of multi-collinearity in subsequent multivariate analyses. The SES index, a composite z-score extracted as the first principal component with highest eigenvalue, was able to sufficiently explain SES of the students (61% of the variance in the measured socio-economic indicators). The respective SES score was categorized into five quintiles (named from low SES level = 1 to high SES level = 5) and used in subsequent regression analyses. The following variables were used in the PCA: mother and father’s educational level (illiterate = 1 to academic = 6), their occupational status (employed vs. unemployed), house ownership (owner vs. tenant), number of rooms per capita, and possession of private room (yes vs. no).

*School characteristics.* In order to investigate the effects of school level characteristics on unhealthy snacking behavior, the following variables were considered: adoption of IHPS program (no vs. yes), school type (public, private), selling snacks at school (yes vs. no), presence of a school health worker (SHW) (yes vs. no), school size (≤150, 151-300, >300), and existence of a space for school canteen (yes vs. no).

Content validity was measured using item-level content validity index (I-CVI)^26^ and Lawshe’s item-level content validity ratio (I-CVR).^27^ The I-CVI for the items ranged from 0.79 to 0.86, with a mean of 0.83; while the I-CVR varied from 0.62 to 0.80, with an average of 0.74. Ten relevant experts (in the fields of nutrition, health education and promotion) participated in validation assessment. The least acceptable critical values for CVI and CVR are considered to be 0.78^26^ and 0.62,^27^ respectively.

Reliability tests of intra-class correlation coefficient (ICC) and Cronbach α were separately calculated for variables when applicable. The test-retest reliability (ICC) for the measures varied from 0.65 to 0.87. The least approvable critical value for ICC is 0.6. All the measures had at least a Cronbach α of 0.6, which is considered acceptable for reliability.^28^ Table 1 illustrates the number of items, reliability tests of each measure, and mean and range of the main measures.

**Table 1:** Cronbach α Coefficient and ICC of the Studied Measures

** Variable**	** No. of items **	** ICC **	** α **	** Mean (Range)**
Cost/price sensitivity	1	0.65	-	2.15 (1-5)
Self-body image	1	0.74	-	3.01 (1-5)
Media advertisements	2	0.87	.78	2.41 (1-5)
Easy accessibility	2	0.69	.71	3.40 (1-5)
Taste and sensory perception	3	0.78	.67	3.50 (1-5)
Perceived self-efficacy	3	0.78	.64	3.60 (1-5)
Perceived parental control	3	0.81	.69	3.20 (1-5)
Nutritional knowledge	4	0.70	.65	2.40 (0-4)
Social norms pressure	7	0.71	.68	3.50 (1-5)

Abbreviation: ICC, Intra-class correlation coefficient.

### Dependent Measure and Instrument

Frequency of unhealthy snacks consumption, as an outcome variable, was the sum of frequencies of consumed unhealthy snacks over a 1-week recall period (7 days prior to the survey day). A 23-item qualitative Food Frequency Questionnaire (FFQ) was used to measure the frequency of unhealthy snacks consumption, as the dependent variable with a count scale (0 = never consumed to 1, 2, 3, …). This questionnaire was firstly obtained from Karimi-Shahanjarini et al study^29^ and then was modified following short semi-structured interviews with fifty students (from middle schools with similar characteristics of the study sample) and common snacks suppliers at 5 school canteens in Kerman city. Face and content validity of the questionnaire were assessed by sending it to 5 nutrition experts. To assure clarity and lack of ambiguity, the questionnaire was then appropriately phrased after distributing it among a sample of students from both IHPS and non-IHPS schools (n = 100) and receiving their feedback. The ICC of the questionnaire was equal to 0.94 after a 2-week interval.

The questionnaire consisted of questions about high-calorie snacks that are low in nutrients and high in fat, sugar, and salt. Snacks were defined as unhealthy according to a national guideline for school canteen foods, jointly issued by the education and health ministries.^30^ Unhealthy snacks were categorized into 3 groups as follows: items with added salt (eg, chips and cheese puffs), with added sugar and high in fat/fast (eg, sugar-sweetened beverages, soft drinks, chocolate cake, gummy candy, jelly sweets, etc), and fast foods (eg, hamburgers, sausages, samosa, falafel, etc).

### Data Collection

Three experienced teams on health surveys were trained in a 1-day workshop to review the objectives and interview techniques of the study. The teams were responsible for data collection. Except for questions about school characteristics which were answered by school principals, the modified qualitative FFQ and all the questions related to the study measures were answered by the students. The randomly selected students from each school were brought together in a class and the questionnaires were distributed among them. In case of the qualitative FFQ, each snack item was read by a facilitator for students to help focus their attention on the proper option. Data were collected over 6 weeks from February to March 2015. All stages of data collection were supervised by the primary researcher.

A written informed consent was obtained from all the respondents and they were free to withdraw from the study whenever they wanted. Parental verbal and informed consent was also obtained through the telephone after choosing the students. In addition, an agreement from authorities and school principals to conduct the research was obtained.

### Data Analysis and Robustness

Descriptive statistics were used to illustrate the general characteristics of schools and students. As the frequency of unhealthy snacks consumption (outcome variable) is a count variable, with an over-dispersion (without zero inflation), negative binomial regression model was used for data analysis. Also, to investigate whether or not students’ unhealthy snacking behavior does vary across school level characteristics, a standard 2-level random intercept (intercepts vary across schools) model was used. As a result, a mixed-effect negative binomial regression model with an independent variance-covariance structure, as a generalization of Poisson model, was used as final multivariate analysis.

To determine the final multivariate model, at first a univariate negative binomial regression analysis was conducted to measure unadjusted effect of factors influencing unhealthy snacking behavior. Then, as presented in Table 2, the variables with a significance level less than 0.20 in the univariate analysis were simultaneously (enter method regression) entered into the multivariate mixed-effect negative binomial regression analysis. Each of the main measures of the study (eg, perceived parental control) was adjusted for other covariates in the model (eg, gender, SES, nutritional knowledge level, media advertisements, perceived self-efficacy, etc).

**Table 2:** Descriptive Summary of Characteristics of Schools (n = 40) and Students (n = 1242)

**Variable**	**Number (%)**
Gender	
Boy	648 (52.20)
Girl	594 (47.80)
Receiving pocket money	
Yes	1018 (82.00)
No	224 (18.00)
Family structure	
Dual-parent	1130 (91.00)
Single-parent/others	112 (9.00)
SES level	
Low	249 (20.05)
Lower middle	248 (19.97)
Middle	249 (20.05)
Higher middle	248 (19.97)
High	248 (19.97)
IHPS program	
Yes	17 (42.50)
No	23 (57.50)
School type	
Public	25 (62.50)
Private	15 (37.50)
Snack sold at school	
Yes	34 (85.00)
No	6 (15.00)
SHW	
Yes	23 (57.50)
No	17 (42.50)
School size	
≤150	14 (35.00)
151-300	15 (37.50)
˃300	11 (27.50)
Presence of a canteen	
Yes	15 (37.50)
No	25 (62.50)

Abbreviations: IHPS, Iranian health promoting school; SES, socio-economic status; SHW, school health worker.

Lowest values of Akaike’s and Schwarz’s Bayesian information criteria measures (AIC and BIC) were used to investigate goodness of fit of the final model. Likelihood ratio test was used as a measure to apply the 2-level negative binomial regression model instead of a negative binomial regression model. The standard residuals analysis was also conducted using the deviance and Pearson methods to better fit the model. Prevalence rate ratios (PRRs) were reported as contributions of independent variables. For categorical variables, the first category was considered as the referent (PRR = 1.00). In continuous variables, the PRR indicates the changes in one unit of the variable. For our non-linear mixed effects models, Spearman or Pearson correlation coefficients with a value greater than 0.50^31^ or variance inflation factor (VIF) higher than 10 were considered as thresholds for multi-collinearity diagnostics test. After exclusion of collinear variables, the remained independent variables were considered as covariates of final model. A robust variance estimator was used to account for the clustering of observations at the student level. Furthermore, no 2-way interaction between independent variables was observed. STATA software version 13.0 (Stat Corp. LP, College Station, TX, USA) was used to analyze the data. To ensure the robustness of the final model, a sensitivity analysis was conducted as follows: unhealthy snacks were disaggregated into three categories of snacks with added fat, salt, and sugar. Then, each category was separately analyzed as a specific outcome.

## Results

Of 1320 questionnaires distributed, 1242 were returned (94.1% response rate). More than half (52.20%) of the participants were boys. 17 schools (42.50%) had already adopted the IHPS program. Nearly two-thirds (62.50%) of schools were public. No snack was being sold in 6 schools (15%). Thus, the students should bring snacks from their home or buy it from shops outside the school. Less than two-thirds (23 schools, 57.50%) of schools had a SHW; 22 schools had a part-time SHW. More than one-third of schools (35.00%) had a population equal or less than 150 students. There was a nutrition canteen in almost one-third of schools (37.50%). Most of students (91%) were living with both parents. The general characteristics of students and schools are presented in Table 2.

For frequency of unhealthy snacks consumption, the findings showed that the average frequency of unhealthy snacks consumed weekly by students was 35.50 (standard deviation [SD] = ±26.0). The highest and lowest frequency of unhealthy snacks consumption per week were related to items with added sugar and added salt reaching to 26.10 (±18.40) and 3.50 (±4.20), respectively. The average frequency of consumption of items high in fat/fast was equal to 5.90 (±7.30) per week.

### Bivariate Analysis

Table 3 illustrates the relationship between independent variables and unhealthy snacks consumption per week in the bivariate analysis. All independent variables, except for presence of a SHW and existence of a school canteen, showed a significance level less than 0.20 in the bivariate analysis. Moreover, a null model of mixed-effect negative binomial regression for unhealthy snacks consumption showed a small but significant raw variance about 20% at the school level. In other words, this raw value was the proportion of total variance in unhealthy snacking behavior between individuals attributable to the school effect.

**Table 3:** Bivariate Analysis Results of Negative Binomial Regression for the Relationship Between the Each Independent Variable With Unhealthy Snacking Behavior

** Independent Variable**	** β Coefficient **	** Unadjusted P Value**
IHPS program (no, yes)	-.10	<.01^a^
School type (public, private)	.12	<.01^ab^
Snack sold at school (no, yes)	-.10	.07^a^
Presence of a SHW (no, yes)	.01	.81
School size (≤150, 151-300, <300)	-.08	<.01^ab^
Presence of a canteen (no, yes)	-.03	.46
Gender (girl, boy)	.11	<.01^a^
SES level (low, lower-middle, middle, higher-middle, high)	.08	<.01^a^
Receiving pocket money (no, yes)	.31	<.01^a^
Family structure (dual-parent, single-parent/others)	.14	.04^a^
Nutritional knowledge	-.08	<.01^a^
Cost/price sensitivity	.03	.08^a^
Taste and sensory perception	.12	<.01^a^
Social norms pressure	.18	<.01^a^
Easy accessibility	.12	<.01^a^
Media advertisements	.06	<.01^a^
Perceived parental control	-.03	.06^a^
Perceived self-efficacy	-.10	<.01^a^
Self-body image	-.05	.05^a^

Abbreviations: IHPS, Iranian health promoting school; SES, socio-economic status; SHW, school health worker. ^a^ Those variables showing a significant level < .2 in bivariate analysis. ^b^ A correlation coefficient higher than 0.50 between school type with school size and IHPS program made a potential risk for severe collinearity and school type was excluded from the multivariate model.

### Multivariate Analysis

Concerning multi-collinearity analysis, the VIF for independent variables ranged from 1.03 to 2.91 with a mean of 1.85. However, a high correlation coefficient between school type with school size (r = 0.71) and IHPS program (r = 0.66) indicated of a potential for multi-collinearity. Hence, the school type was excluded from the final model to better fit the model for estimation.

As shown in Table 4, with simultaneous adjustment for covariates in the final model, being boy (adjusted PRR = 1.20; 95% CI: 1.05-1.38) and taste and sensory perception (adjusted PRR = 1.18; 95% CI: 1.09-1.27), as intrapersonal factors, had significant adjusted associations with the higher weekly consumption of unhealthy snacks. Moreover, better nutritional knowledge level (adjusted PRR = 0.96; 95% CI: 0.91-0.99) about snacks had also an adjusted association with lower frequency of unhealthy snacks consumption after controlling for the covariate factors in the final model. The higher perceived self-efficacy (adjusted PRR = 0.95; 95% CI: 0.91-1.00) for unhealthy snacks consumption had a similar result, but with this approaching significance (*P* < .06).

**Table 4:** Final Multivariate Model of Factors Affecting the Unhealthy Snacking Behavior in Adolescents Using Mixed-Effect Negative Binomial Regression Analysis

** Variable**	** Frequency Consumption of Unhealthy Snacks **		
	** β Coefficient **	** Robust Standard Error**	** Adjusted PRRa (95% CI)**
IHPS program			
No	Reference	-	Reference
Yes	.05	0.07	1.05 (0.91-1.22)
Gender			
Girl	Reference	-	Reference
Boy	.18	0.07	1.20 (1.05-1.38)^b^
SES level			
Low	Reference	-	Reference
Lower-middle	.08	0.04	1.08 (0.98-1.19)
Middle	.24	0.06	1.27 (1.11-1.45) ^b^
Higher-middle	.36	0.05	1.45 (1.30-1.62) ^b^
High	.37	0.07 1.45 (1.26-1.67) ^b^
Pocket money allowance			
No	Reference	-	Reference
Yes	.19	0.06	1.21 (1.09-1.34) ^b^
Family structure			
Dual-parent	Reference	-	Reference
Single-parent/others	.13	0.06	1.14 (1.01-1.30) ^b^
School size			
≤150	Reference	-	Reference
151-300	-.02	0.07	0.97 (0.84-1.11)
˃300	-.23	0.07	0.79 (0.68-0.92) ^b^
Snack sold at school			
No	Reference	-	Reference
Yes	.01	0.08	1.01 (0.85-1.19)
Nutritional knowledge	-.04	0.02	0.96 (0.91-0.99) ^b^
Cost/price sensitivity	.01	0.02	1.00 (0.96-1.04)
Taste and sensory perception	.16	0.03	1.18 (1.09-1.27) ^b^
Social norms pressure	.08	0.03	1.08 (1.01-1.17) ^b^
Easy accessibility	.06	0.02	1.06 (1.01-1.11) ^b^
Media advertisements	.01	0.02	1.01 (0.97-1.05)
Perceived parental control	-.04	0.02	0.96 (0.92-0.99) ^b^
Perceived self-efficacy	-.04	0.02	0.95 (0.91-1.00) ^c^
Self-body image	-.03	0.02	0.96 (0.92-1.01)
*Constant*	*2.54*	*0.23*	*12.90 (8.12-20.01)*
*Variance of school level*	*.01*	*0.00*	-
*Variance of school level (null model)*	*.02*	-	-
*Wald chi2 (P value); df = 20*	*376 (< .01)*	-	-

Abbreviations: IHPS, Iranian health promoting school; SES, socio-economic status; PRR, prevalence rate ratio. ^a^ Enter method regression was used for the multivariate analysis. Adjusted PRR for each independent variable of the full final model by holding all the other covariates constant. ^b^ Statistically significant at P < .05. ^c^ Statistically significant at P < .10.

Students with middle, higher-middle, and high SES levels had higher frequency of unhealthy snacks consumption (27%, 45%, and 45%, respectively) after keeping all the covariates constant. Similarly, after controlling for the listed covariates in Table 4, higher social norms pressure (adjusted PRR = 1.08; 95% CI: 1.01-1.17), single-parent family (adjusted PRR = 1.14; 95% CI: 1.01-1.30), less perceived parental control (adjusted PRR = 0.96; 95% CI: 0.92-0.99), receiving pocket money (adjusted PRR = 1.21; 95% CI: 1.09-1.34), and easier accessibility to unhealthy snacks (adjusted PRR = 1.06; 95% CI: 1.01-1.11) were significantly associated with higher weekly consumption of unhealthy snacks. It was also observed that when adjusted for the covariates, schools with a size larger than 300 students had lower weekly consumption of unhealthy snacks compared to small schools with less than 150 students (adjusted PRR = 0.79; 95% CI: 0.68-0.92)**.**

In the final model of the multivariate analysis, it was also found that after controlling for other factors in the final model, there was no adjusted association between IHPS program adoption, self-body image, accessibility of snacks at school, media advertisements, and lower cost of unhealthy snacks with unhealthy snacking behavior (*P* > .10; Table 4).

The results of sensitivity analysis showed that although running the final models using subcategories of unhealthy snacks led to small changes in coefficients of predictors, but it did not influence the statistical significance of the effects.

## Discussion

The present study attempted to investigate factors that affect unhealthy snacking behavior among adolescents in Kerman city, Iran. The findings of our study support this notion that health-related behaviors are highly multi-dimensional and complicated. Therefore, in order to change such a health behavior, addressing a wide range of different individual, socio-cultural, and physical-environmental factors is necessary. This requires adoption of sound interventions supported and underpinned by evidence about effects of those determinants. This study offers some important insights about factors influencing consumption of unhealthy snacks. In fact, the findings of the present study have a number of practical implications to better develop school-based interventions, especially for ongoing IHPS program.

Considering mixed findings of previous studies, a controversial matter is the extent that schools are affecting dietary behaviors in Iran. It was found in the present study that variation between schools after adjusting for individual and environmental factors was small but significant. Additionally, among the school level variables, schools with a larger size had an effect on lower consumption of unhealthy snacks. This finding can be explained by this fact that small schools are mostly either private schools with high SES level or schools that lack a SHW, so less supervised and sensitive about snacks sold at canteens. Moreover, after adjusting for covariates, other school level variables including adoption of IHPS program and selling snacks at school were not associated with unhealthy snacking behavior. This issue might be due to misunderstanding of HPS philosophy and also of inadequate advocacy of national and local authorities for institutionalization of IHPS program in the selected schools. A study among Iran’ schools in 2015 showed that IHPS schools rarely afford to provide a healthy food environment better than that of non-IHPS schools (V. Yazdi Feyzabadi, N. Omidvar, N. Keshavarz Mohammadi, S. Nedjat, A. Karimi-Shahanjarini, A. Rashidian, unpublished data).

In bivariate analysis, findings showed that attendance of a SHW had no association with unhealthy snacks consumption. It might be a result of this issue that there is a shortage of SHWs in Iran’s schools, leading to part-time attendance of SHWs at schools. This matter might lead to intermittent supervision of SHWs on snacks provision and consumption at schools and less opportunity to make sustained changes in healthy food environment. In a study by Gugglberger and Dür among school heads in Austria, it was concluded that knowledge management and financial and human resources are key factors for effective implementation of health promotion interventions in schools.^32^

Researchers have already drawn attention to effects of schools on students’ health behaviors. In a longitudinal study with over 2000 adolescents in Scotland, West et al concluded that schools have some effects on students’ health behaviors, except for dietary behaviors which are mostly shaped in earlier stages of life influenced by SES and cultural characteristics.^33^ Interestingly, some systematic reviews have indicated that there is either insufficient and limited^34,35^ or no evidence for effect^36^ of school-based interventions that mainly focus on multi-components (education and environment changes) to change dietary intake among adolescents, particularly among those aged 13-18 years old. However, few studies have concluded that school-based interventions, particularly HPS, have minimal to moderate effect on healthy and unhealthy snacks consumption.^10,37^ This matter invites for future research on this issue, particularly among students with higher age.

Findings revealed that gender, SES, taste and sensory perception, receiving pocket money, family structure, school size, social norms pressure, easy accessibility, parental control, self-efficacy, and nutritional knowledge affect the unhealthy snacks consumption among adolescents. Similarly, results of a qualitative study conducted in Tehran city in 2008 showed that taste, peer pressure, parental influence, easy access to unhealthy snacks, and appeal of snacks had an effect on unhealthy snacks consumption.^38^ These factors might be modifiable if school-based dietary interventions along with multi-component interventions, particularly HPS approach, address the factors in an effective partnership with stakeholders within and without the school community.^39^

Another important finding was that students with higher SES had higher consumption of unhealthy snacks. In line with this finding, a study carried by Shi et al in China showed that SES was positively and significantly associated with intake of fast foods and soft drinks.^40^ Another study also highlighted that school-based dietary interventions had different effects across SES subgroups.^15^ Our results also revealed that there was a gender difference between adolescents in unhealthy snacks consumption, indicating that boys consumed unhealthier snacks. This is consistent with findings of the studies conducted by Park et al in the United States in 2010 and Wouters et al in the Netherlands in 2008.^41,42^ It can be then advised that schools with HPS should develop and designate dietary interventions consistent with gender and SES differences. This may make such interventions more effective.

Interestingly, the pressure emanated from social norms, such as unhealthy snacks consumption by peers and family members, and also society’s expectations stimulated higher consumption of unhealthy snacks in our study. This matter, however, is in line with other studies.^42,43^

Students with higher self-efficacy and higher nutritional knowledge had lower consumption of unhealthy snacks. This is consistent with findings of the studies conducted by Fitzgerald et al^44^ in Ireland in 2009 and Grosso et al in Italy in 2010.^45^ Moreover, other studies have also shown that the role of adolescents’ peer networks and peer-led education programs might be important for promotion and maintenance of positive eating behaviors.^46,47^ In a study conducted in China in 2012-2013, it was found that school-based nutrition education programs increase healthy foods intake.^48^ Thus, the programs that aim to change self-efficacy, integrate nutritional knowledge into school curriculum, and include peer support and education programs to limit unhealthy snacks consumption and promote healthy snack options, may lead to improvement of snacking behaviors among adolescents.

Media advertisements had no adjusted association with unhealthy snacking behavior. This matter can be due to limited number of items under the measure in our study. A study in Australia in 2009-2010 showed that in addition to television food advertising, digital food marketing and print/transport/school food marketing were also associated with unhealthy snacks consumption.^49^ It seems, however, that the adolescents are more exposed to video games and mobile-based internet sources which were not included in this study.

Moreover, better taste and appeal of unhealthy snack were related to higher consumption. This was consistent with results of other studies in Iran in 2008 and in Turkey in 2003-2004 in which taste and sensory perception acted as determinants of food choice among the adolescents.^38,50^ This matter might be a result of policies adopted by food industries.

Easy access to unhealthy snacks at home and food stores along with less parental control had an effect on higher unhealthy consumption. This was consistent with findings of Campbell et al in Australia in 2002-2003; in their longitudinal study it was found that availability of unhealthy foods at home was positively associated with unhealthy snacking behavior among adolescents.^51^

The findings also showed that parenting practices to control consumed snacks reduced the frequency of unhealthy snacks consumption. A study among Iranian female adolescents in Tehran city reported of similar parental effect on junk food consumption.^29^ Accordingly, it can be suggested that schools should use the existing institutions such as parents and teachers associations within schools to enhance partnership between parents and schools and thereby restrain unhealthy snacks consumption and provide healthier food environment for adolescents. A systematic review showed that in HPS schools where there was a strong engagement between parents and communities at different levels, a more supportive environment for adoption of a healthy diet was provided.^10^ Furthermore, a meta-analysis showed that parental or family involvement in nutritional interventions at schools decrease weight gain^52^ and provide students with healthier food options.^53^ This matter points to considerable role of family and community engagement in helping schools to adopt a set of health policies that encourage limited access to unhealthy snacks at home through systematic partnership of parents and students.

## Study Limitations

The present study had number of limitations. First, as this was a cross-sectional study, direct causality inference of findings should be made cautiously. Second, the adolescents participated in this study were only students from eighth grade, so they were not a representative sample of Iranian adolescents. This limits the generalizability of the ﬁndings. Third, low precision of the measures, particularly those with one or a few questions, might be the reason for small effects of some factors (eg, price, taste and sensory perception, media, nutritional knowledge, and self-efficacy). However, content validity (CVI and CVR) and reliability of the measures (ICC and Cronbach α) were more or less acceptable. Fourth, a self-reported questionnaire with 1-week recall period were used to measure unhealthy snacking frequencies. This type of assessment, however, may be influenced by recall and reporting biases. Additionally, although unhealthy snacking behavior was assessed by relevant questions from a validated FFQ in a study conducted on Iranian adolescents, the validity of the FFQ might be affected by some revisions that were made to ensure the representativeness of items in the participants’ diets. Moreover, although the modified qualitative FFQ was validated using experts’ opinion and short interviews with local students, dietary data were not assessed using identical versions of the FFQ administered within a 24-hour recall interval. However, the ICC of modified FFQ was sufficiently good. Fifth, this study was unable to encompass all factors affecting unhealthy snacking, but most common factors in the intrapersonal and environmental levels were included. Finally, the major limitation of multi-level analysis is sample size of higher level (here school level). Thus, as a larger sample size in higher level would result in an unbiased estimation of the second-level standard errors,^54^ the number of schools maybe be also a matter of concern in our study. Thus, we recommend conducting further research with larger sample size in higher level (number of schools) to attain more definite results.

## Conclusion

Our findings might provide implications for policy-makers at national and local levels in order to effectively develop IHPS program. There was an array of individual, socio-cultural, and physical environmental factors that affected unhealthy snacks consumption among adolescents in Kerman. In addition, it was revealed that school level features had a small effect on unhealthy snacks consumption. An important practical implication of the findings is that effects of school-based initiatives can be improved by focusing on policies and interventions aimed at determinants of unhealthy snacking behavior. These policies and interventions should particularly focus on role of parents, self-efficacy, nutritional knowledge, SES level, and social norms. It is suggested that the schools, particularly IHPS ones, should adopt multi-component approaches that combine school curriculum and environmental changes in a systematic manner. This should be in partnership with parents, students, school staff, and wider community to promote healthy food options and limit unhealthy snacks.

## Acknowledgments

This study is part of a PhD thesis in Health Policy. This study was supported by Tehran University of Medical Sciences (TUMS), Tehran, Iran with grant number 9021460007. We are grateful to the adolescents who willingly participated in this study. Authors also would like to thank all the staff members of schools, authorities of education sector and questioning teams for their cooperation in this study.

## Ethical issues

Ethics committee of Tehran University of Medical Sciences (TUMS), Tehran, Iran approved the study protocol with ethical approval code: 1394.833.

## Competing interests

Authors declare that they have no competing interests.

## Authors’ contributions

VYF was involved in the study design, data collection, data entry, analysis, and drafted the original manuscript. AR was involved in the study design, the statistical analysis, and drafting the manuscript. NKM and NO contributed to the study design particularly the questionnaire development. AKS also contributed to study design and some modifications in the questionnaire. SN contributed to some statistical consultations. All authors read and approved the final manuscript.

## Authors’ affiliations

^1^Department of Health Management and Economics, School of Public Health, Tehran University of Medical Sciences, Tehran, Iran. ^2^School of Public Health, Shahid Beheshti University of Medical Sciences, Tehran, Iran. ^3^Department of Community Nutrition, National Nutrition and Food Technology Research Institute, Faculty of Nutrition Sciences and Food Technology, Shahid Beheshti University of Medical Sciences, Tehran, Iran. ^4^Social Determinants of Health Research Center and Department of Public Health, School of Public Health, Hamadan University of Medical Sciences, Hamadan, Iran. ^5^Epidemiology and Biostatistics Department, School of Public Health, Knowledge Utilization Research Center, Tehran University of Medical Sciences, Tehran, Iran.

## Key messages

**Implications for policy makers**

- Different individual, socio-cultural and physical-environmental influences affect unhealthy snacks consumption among adolescents in Iran.
- Parenting practices towards the limited unhealthy snack intake, peer unhealthy snacks consumption, more accessibility to unhealthy snacks at shops and home, less knowledge about healthy and unhealthy snacks, and lower self-efficacy to limit the consumption of unhealthy snacks affect higher consumption of unhealthy snacks. Adoption of supportive strategies to raise participation at home and school in educational programs such as peer education and social networks, and to increase the collaborative role of communities in creating healthy food environments can be beneficial for restraining unhealthy snacks consumption.
- School size as a school characteristic is inversely associated with unhealthy snacking behavior.
- It might be useful if schools, through reorientation of student and parent boards and councils, could develop promising school policies and practices focusing on gender and socio-economic factors, school curriculum, extra-curriculum, and after-school activities to create a healthy food environment.

**Implications for public**

Non-communicable Diseases (NCDs) are one of the main global public health problems, particularly in developing countries. These diseases stem from high-risk behaviors which are mainly shaped or intensified during childhood and adolescence. Unhealthy snacking is one of those behaviors which have recently been addressed by establishment of Iranian Health Promoting School (IHPS) program. In the present study, we aimed to investigate the factors that affect consumption of unhealthy snacks (those high in calorie, fat, sugar, and salt) among Iranian adolescents and provide appropriate evidence to strengthen the IHPS program. Our findings showed that unhealthy snacking behavior is mainly affected by poor parenting practices towards the limited unhealthy snack intake, family conditions particularly higher socio-economic status (SES), easy access to unhealthy snacks at shops and home, smaller school size, and limited empowerment of the students. It is therefore suggested that schools (particularly those with IHPS) should, in collaboration with students, parents and community, focus on adoption of strategies that promote healthy food choices and restrain unhealthy food access.
